# Metabolic syndrome is associated with a poor outcome in patients affected by outflow tract premature ventricular contractions treated by catheter ablation

**DOI:** 10.1186/1471-2261-14-176

**Published:** 2014-12-06

**Authors:** Celestino Sardu, Giovanni Carreras, Spyridon Katsanos, Vasileios Kamperidis, Maria Caterina Pace, Maria Beatrice Passavanti, Ilaria Fava, Pasquale Paolisso, Gorizio Pieretti, Giovanni Francesco Nicoletti, Gaetano Santulli, Giuseppe Paolisso, Raffaele Marfella

**Affiliations:** Department of Medical, Surgical, Neurological, Metabolic and Geriatric Sciences, Second University of Naples, Naples, Italy; Cardiovascular Department and Electrophysiology Unit, Santa Maria Terni Hospital, Terni, Italy; Cardiology, KAT hospital, Athens, Greece; 1st Cardiology Department, AHEPA University Hospital, Thessaloniki, Greece; Department of Anesthesiological Surgical and Emergency Sciences II, University of Naples, Naples, Italy; Department of Plastic and Reconstructive Surgery, Second University of Naples, Naples, Italy; Columbia University Medical Center, New York, New York, USA; Piazza Miraglia, 2, Napoli, 80138 Italy

## Abstract

**Background:**

The purpose of this study was to investigate the impact of metabolic syndrome (MS) on outcome of catheter ablation (CA) for treatment of frequent premature ventricular contraction beats (PVCs) originating from right ventricular outflow tract (RVOT), left ventricular outflow tract (LVOT) or coronary cusps (CUSPs), in patients with normal ventricular systolic function and absence of cardiac structural disease.

**Methods:**

In this multicentre prospective study we evaluated 90 patients with frequent PVCs originating from RVOT (n = 68), LVOT (n = 19) or CUSPs (n = 3), treated with CA. According to baseline diagnosis they were divided in patients with MS (n = 24) or without MS (n = 66). The study endpoint was a composite of recurrence of acute or delayed outflow tract ventricular arrhythmia: acute spontaneous or inducible outflow tract ventricular arrhythmia recurrence or recurrence of outflow tract PVCs in holter monitoring at follow up.

**Results:**

Patients with MS compared to patients without MS showed a higher acute post-procedural recurrence of outflow tract PVCs (n = 8, 66.6%, vs. n = 6, 9.0%, p = 0.005). At a mean follow up of 35 (17-43) months survival free of recurrence of outflow tract PVCs was lower in patients with baseline MS compared to patients without MS diagnosis (log-rank test, p < 0.001). In cox regression analysis, only MS was independently associated with study endpoint (HR = 9.655 , 95% CI 3.000-31.0.68 , p < 0.001).

**Conclusions:**

MS is associated with a higher recurrence rate of outflow tract PVCs after CA in patients without structural heart disease.

## Background

According to the Third report of the National Cholesterol Education Program (NCEP) expert panel on detection, evaluation, and treatment of high blood cholesterol in adults (Adult Treatment Panel III) [1], metabolic syndrome (MS) is a clinical condition defined by evidence of several risk factors including obesity, hypertension, diabetes, and dyslipidaemia and it is related to a pro inflammatory and pro thrombotic state [2]. MS identifies patients at increased risk for cardiovascular disease (CVD), type 2 diabetes mellitus, and all-cause mortality [3–5], and approximately one-fifth of the adult U.S. population would be classified as having MS with an upward trend in abdominal obesity and insulin resistance [6]. Patients with uncomplicated MS have a greater dispersion of ventricular repolarization time and augmented maximal and minimal QTc [7], with increased possibility of ventricular re-entry circuits [8–10] and increased frequency of premature ventricular contractions (PVCs) compared to healthy controls [11, 12]. Ventricular arrhythmias and PVCs originating from right ventricular or left ventricular outflow tract (RVOT, LVOT) and coronary cusps (CUSPs), characterized by inferior axis, precordial left bundle branch block (LBBB) morphology and V3 or V4 precordial transition on surface electrocardiogram (ECG) are often benign and not related to structural heart disease [13]. These arrhythmias are caused by triggered activity due to cyclic adenosine monophosphate (cAMP)- mediated calcium-dependent delayed after depolarizations, and related to a arrhythmogenic cell focus firing and triggering induction mechanisms like catecholamine infusion, and are responsible to adenosine as well as verapamil administration [14]. Recent data evidenced the role of oxidative stress in initiating cardiovascular consequences in MS. The enhanced oxidative stress exacerbates inflammation, which in turn further exacerbates oxidative stress, generating a vicious cycle, leading to sympathetic over activity and eventually ventricular arrhythmias [15]. Thus, sympathetic over activity state related to MS may represent an irritating and triggering mechanism on these outflow tract PVCs. According to guidelines there is a class I indication for ventricular arrhythmias catheter ablation (CA) in patients at low risk for sudden death, in absence of structural heart disease [16] and authors have observed beneficial effects on left and right ventricular function in patients with PVCs and preserved left ventricular ejection fraction [17]. There are no evidences about catheter ablation (CA) outcomes in MS patients affected by idiopathic or benign PVCs. In this context, our study hypothesis is to examine the impact of MS on CA outcome of idiopathic PVCs in patients with preserved ventricular systolic function and absence of cardiac structural disease.

## Methods

### Patient population

In this multicentre prospective study we evaluated, from September 2010 to December 2013, all consecutive patients (n = 90) who were treated by CA of idiopathic focal PVCs, originating from the RVOT (n = 68), LVOT (n = 19) or CUSPs (n = 3), at Federico II university of Naples (Naples, Italy), S. Maria di Loreto Mare Hospital (Naples, Italy), and S. Maria Terni Hospital (Terni, Italy). Inclusion criteria were: 1) presence of RVOT, LVOT or CUSPs origin PVCs, 2) all patients have discontinued the anti-arrhythmic drugs (AADs) 4-5 half live before to perform the ablation, 3) absence of any structural heart disease, normal ventricular systolic function, 4) normal thyroid function and electrolyte values, 5) normal renal function, 6) absence of neoplastic disease. For every patient to rule out structural heart disease, a comprehensive clinical evaluation was performed, which included medical history, 12-lead electrocardiogram (ECG), and two dimensional transthoracic echocardiography. If exclusion of structural heart disease was deemed necessary, exercise testing, coronary angiography, cardiac multidetector computed tomography (CMCT), and/or nuclear imaging were performed. Prior to CA, 12-lead ECG recording of the PVCs and 24-hour Holter registration were acquired for all patients, and the evidence of more than three consecutive monomorphic PVCs beats were classified as ventricular tachycardia (VT). Baseline fasting blood samples were obtained from all patients for the measurement of blood glucose, lipid profile, and C Reactive Protein (CRP) (Elecsys 2010, Roche Diagnostics). The CRP cut off value was set at 0.9 mg/dl, with values below and above this cut off considered normal and high, respectively. Nitrotyrosine plasma concentration was assayed by enzyme-linked immunosorbent assay. Nitrotyrosine was determined because this modified amino acid is a product of free-radical (O^2–^) interaction with nitric oxide (NO). The interaction of O^2–^ with NO leads to a rapid inactivation of NO and to a production of the potent oxidant peroxynitrite. Detection of nitrotyrosine is strongly suggestive of increased generation of peroxynitrite [18]. Patients were diagnosed with MS if they had at least 3 of these risk factors, according to Adult Treatment Panel III MS definition [1]: waist circumference >102 cm in men and 88 cm in women, high-density lipoprotein <40 mg/dl in men and <50 mg/dl in women and serum triglycerides >150 mg/dl, blood pressure >130/85 mm Hg or on antihypertensive medication, fasting blood glucose >100 mg/dl or on antidiabetic medication. According to authors [19] if body mass index is over 30 kg/m2, central obesity can be assumed and waist circumference does not need to be measured. The study was approved by institutional review boards and ethics committees at Federico II university of Naples (Naples, Italy), S. Maria di Loreto Mare Hospital (Naples, Italy), and S. Maria Terni Hospital (Terni, Italy). All patients provided written informed consent.

### Ablation procedure

In discontinuation of AADs 4-5 half live before performing the electrophysiological study and catheter ablation, and in conscious sedation by transfemoral right femoral vein access a quadripolar diagnostic catheter 4 mm fixed curve (Biosense Inc., Diamond Bar, CA; St. Jude Medical, Inc., St. Paul, MN, USA ) has been placed first in right ventricle apex and subsequently in RVOT for reference and pacing manoeuvres. In the presence of clinical PVCs, activation mapping was performed using a 4-mm tip ablation catheter, placed by transfemoral right femoral vein (RVOT) or artery (LVOT and CUSPs) and pace mapping was performed in addition to activation mapping to identify the PVC focus during sinus rhythm. In patients without spontaneous PVCs, programmed ventricular stimulation was performed from the right ventricular (RV) apex and RVOT at three drive cycle lengths with up to three extra stimuli and incremental burst pacing at a cycle length up to 250 ms, at baseline and during isoproterenol infusion. All endocavitary signals have been registered, filtered and analysed by an expert cardiologist by a polygraph (Lab System Pro, Bard Electrophysiology, Lowell, MA, USA). In all patients, a three-dimensional non-fluoroscopic imaging and mapping system (CartoXP and Carto 3, Biosense Inc., Diamond Bar, CA; Ensite Navx, St. Jude Medical, Inc., St. Paul, MN, USA) was used for catheter localization and activation mapping. Radiofrequency energy was delivered with a 4-mm-tip non irrigated catheter (Biosense Inc., Diamond Bar, CA, or St. Jude Medical, Inc., St. Paul, MN, USA ) with a target temperature of 60°C at a power of 35 W or with a 4-mm-tip irrigated ablation catheter (Biosense Inc., Diamond Bar, CA or St. Jude Medical, Inc., St. Paul, MN, USA) in temperature-controlled mode with a target temperature of 45°C at a power of 30 W. If the PVCs were abolished within 20 seconds, the energy application was continued for 60 seconds, while if PVCs were still present after 25-30 seconds, the energy application was terminated, and mapping was continued to find an optimal target site. After catheter ablation programmed ventricular stimulation performed from the right ventricular (RV) apex and RVOT at three drive cycle lengths with up to three extra stimuli and incremental burst pacing at a cycle length up to 250 ms, at baseline and during isoproterenol infusion. On and off isoproterenol infusion were utilized to confirm the effectiveness of CA in all patients. Acute success was defined as the absence of out flow tract PVCs with similar morphology during a 30 minutes observation period after CA, and in absence of clinical PVCs during programmed ventricular stimulation.

### End point

The end point was considered a composite of 1. acute absence of spontaneous or inducible outflow tract PVCs (OT-PVCs), with isoproterenol infusion or bursts pacing from right ventricular apex and RVOT for 30 minutes following the last radiofrequency lesion, and 2. delayed and late recurrence of OT-PVCs at a rate of ≤ 300 beats per day documented by 24 h Holter monitoring at scheduled follow up visits.

### Follow up

All patients were followed in the outpatient department within 2 weeks, and thereafter at monthly intervals, during which physical examination and 12-lead ECG were conducted. Routine 12-lead Holter monitoring was performed at the 1^st^, 3^rd^, 6^th^ and 12^th^ month and echocardiography was performed at the 3^rd^ and 6^th^ month floow-up. Whenever patients had symptoms of palpitations, dizziness, or syncope during follow-up, they were advised to contact their doctors immediately for evaluation of vital signs, 12-lead ECG, and12-lead 24 hour Holter monitoring. All AADs were discontinued after successful ablation procedure.

### Statistical analysis

Statistical analyses were carried out with a package of SPSS software version 20 (SPSS Inc., Chicago, IL, USA). Kolmogorov-Smirnov test was used to check the Gaussian distribution of continuous variables. Continuous variables were presented as mean ± standard deviation if normally distributed or as median (interquartile range), otherwise. Categorical variables were presented as number and frequencies. Patients were dichotomized according baseline diagnoses of MS. The Student’s t-test or Mann-Whitney test were used to compare continuous variables as appropriate. Additionally, χ^2^ test or Fisher’s exact test, were used to compare categorical variables, as recommended. All long term survival analyses were calculated from the date of the procedure. The Log-rank test for time-to-event data with respect to recurrence of study endpoint was used for statistical comparison between patients with and patients without MS. Univariate and multivariate Cox proportional hazards models were performed to identify independent associates of the endpoint. The estimated hazard ratios and the 95% confidence intervals were computed. Only univariate variables with p <0.05 were included in the multivariate model. For multivariate modelling, BMI > 30 kg/m^2^, hypertension, dyslipidaemia and diabetes were omitted due to collinearity with MS. A two-sided p <0.05 was considered statistically significant.

## Results and discussion

The mean age of the population was 40 ± 16 years (male 53.3%). By definition, patients with MS had higher BMI, and higher rates of dyslipidaemia, hypertension, and diabetes (Table 1). Creatinine, Nitrotyrosine and C Reactive Protein (CRP) were also higher in the MS group (1.1(1.0-1.2) mg/dl vs 1.0 (0.9-1.0) mg/dl, p = 0.004, 0.42 ± 0.03 μmol/L v/s 0.27 ± 0.02 μmol/L, p < 0.001 and 5.3 (3.2-7.2) mg/dl, vs. 2.7 (1.7-4.0) mg/dl, p < 0.001, respectively). Values of oxidative stress marker (nitrotyrosine) levels were higher in MS group (Table 1). Patients with MS compared to patients without MS were more commonly treated with angiotensin-converting enzyme inhibitors and angiotensin receptor blockers (ACE/ARB blockers), beta-blockers and lipid-lowering therapy (Table 1). Interestingly, patients with MS as opposed to patients without MS had higher baseline heart rate (HR) (72 ± 11 beats/min vs. 65 ± 10 beats/min, p = 0.015), higher PVCs burden at baseline (7263 ± 1766 beats/day vs 7263 ± 1766 beats/day, p = 0.042) and longer CA procedural time (163.9 ± 39.1 min vs. 141 ± 24 min, p = 0.002) (Table 2).

**Table 1:** **Baseline population characteristics**

Variable	Total population	Patients with MS	Patients without MS	p-value
	(n = 90)	(n = 24)	(n = 66)	
Age (years)	40 ± 16	53 ± 12	35 ± 15	<0.001
Male n(%)	48 (53.3%)	10 (41.6%)	38 (57.5%)	0.181
BMI (kg/m^2^)	27.7 ± 3.3	31.7 ± 1.2	26.3 ± 2.6	<0.001
Dyslipidemia n (%)	32 (35.6%)	22 (91.6%)	10 (15.1%)	<0.001
Hypertension n (%)	41 (45.6%)	23 (95.8%)	18 (27.2%)	<0.001
Diabetes n (%)	10 (11.1%)	10 (41.6%)	0 (0%)	<0.001
Creatinine (mg/dl)	1.0 (0.9-1.2)	1.1(1.0-1.2)	1 (0.9-1.0)	0.004
Nitrotyrosine (μmol/L)	0.31 ± 0.07	0.42 ± 0.03	0.27 ± 0.02	<0.001
CRP (mg/dl)	3.7 (1.8-5.3)	5.3 (3.2-7.2)	2.7 (1.7-4.0)	<0.001
**PVCs origin site**				
RVOT n (%)	68 (75.6%)	20 (83.3%)	48 (72.7%)	0.791
LVOT n (%)	19 (21.1%)	3 (12.5%)	16 (24.2)	0.227
CUSPs n (%)	3 (3.3%)	1 (4.1%)	2 (3.0%)	0.610
**Echocardiography**				
LVTDd (mm)	50.0 ± 5.5	50.1 ± 5.3	50.0 ± 5.5	0.933
LVTSd (mm)	28.7 ± 4.6	30.2 ± 4.3	28.1 ± 4.6	0.058
LVEF (%)	57 ± 5	57 ± 3	57 ± 5	0.982
**Medication**				
ACEi/ARBs n (%)	33 (36.7%)	21 (87.5%)	12 (18.1%)	<0.001
Beta blockers n (%)	16 (17.8%)	13 (54.1%)	3 (4.5%)	<0.001
Lipid-lowering therapy n (%)	29 (32%)	19 (79.1%)	10 (15.1%)	<0.001
Class I AADs n (%)	16 (17.8%)	11 (45.8%)	5 (7.5%)	<0.001
Class III AADs n (%)	3 (3.3%)	2 (8.3%)	1 (1.5%)	0.172
Class IV AADs n (%)	3 (3.3%)	2 (8.3%)	1 (8.3%)	0.172
Follow-up months n (%)	39 (32-45)	35 (24-45)	40 (34-45)	0.356

Continuous variables are expressed as mean ± SD if normally distributed or as median (IQR: 25th percentile, 75th percentile) if not normally distributed, categorical variables are expressed as number (percentage). AADs anti arrhythmic drugs; ACEi/ARBs blockers angiotensin-converting enzyme inhibitors and angiotensin receptor blockers; CRP C-reactive protein; CUSPs aortic cusps; LVEF left ventricle ejection fraction; LVOT left ventricular outflow tract; LVTDd left ventricle end-diastolic diameter; LVTSd left ventricle end-systolic diameter; PVCs premature ventricular contractions; RVOT right ventricular outflow tract.

**Table 2:** **HR, PVCs morphology, PVCs burden and ablation procedure characteristics**

Variable	General population	Group 1 (with MS)	Group 2 (without MS)	P Value
		(n = 24)	(n = 66)	
HR baseline (bpm)	67 ± 10	72 ± 11	65 ± 10	0.015
PVC burden	7455 ± 2021	8226 ± 2 407	7263 ± 1766	0.042
QS wave in DI n (%)	23 (25.6%)	4 (16.6%)	19 (28.7%)	0.244
Precordial transition before V4 n (%)	11 (12.2%)	2 (8.3%)	9 (13.6%)	0.721
Fluoroscopic time (min)	22.8 ± 10.2	22.7 ± 8.0	20.5 ± 9.1	0.299
RF time (min)	6.54 ± 3.4	7.2 ± 2.8	6.2 ± 3.5	0.206
Procedural time (min)	147.55 ± 30.8	163.9 ± 39.1	141 ± 24	0.002

Variables are expressed as mean ± SD or median (IQR: 25th percentile, 75th percentile) if not normally distributed.

AADs, antiarrhythmic drugs; HR, heart rate in beats for minute (bpm); PVC, premature ventricular beats; QS wave in DI derivation on surface ecg; RF, radiofrequency catheter ablation.

### Study endpoint

There were 14 cases of acute PVCs recurrence (15,5%). The rate of spontaneous or inducible PVCs with isoproterenol infusion and bursts pacing from right ventricular apex and RVOT for 30 minutes following the last radiofrequency lesion acute PVC was significantly higher in patients with MS compared to patients without MS (n = 8, 66.6%, vs. n = 6, 9.0%, p = 0.005). During a follow up there were, 8 more patients (8,8%), evidenced of PVCs at a rate of > 300 beats per day documented by 24 h Holter. In total, 22 (24.4%) patients reached the study endpoint (14 patients had acute and 8 patients delayed PVCs). None of the patients died during follow-up.

**Figure 1:**
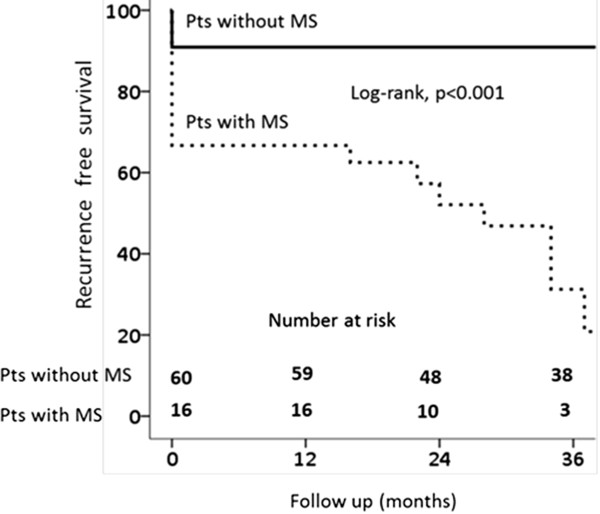
**Recurrence-free survival in patients with PVCs.** Kaplan Meier curve showing survival free of study endpoint between patients with or without metabolic syndrome (Log-rank test, p < 0.001). MS = metabolic syndrome; PVCs = outflow tract premature ventricular contractions; Pts = patients.

The Kaplan–Meier curves show survival rates free of the composite study endpoint for patients divided according to baseline diagnosis of MS (Figure 1). The recurrence of acute or delayed PVCs free survival at 12 and 24 months follow up in the MS group were 50% and 9% compared with 89% and 87% in the group of patients without MS, respectively (log-rank p < 0.001) (Figure 1).

From the univariate Cox proportional hazards analysis, MS (HR = 9.119, 95% CI =3.461-24.024, p < 0.001), CRP (HR = 1.253 95% CI = 1.035-1.516, p = 0.021), and procedural time (HR = 1.015, 95% CI = 1.004-1.027, p = 0.015) were included in the multivariable model (Table 3). Finally, only MS was independently associated with the study endpoint (HR = 9.119, 95% CI = 3.461-24.024, p < 0.001) (Table 3).

**Table 3:** **Multivariate cox regression analysis for parameters associated with study endpoint**

Variable	Univariate analysis		Multivariate analysis	
	HR (95% CI)	p-value	HR (95% CI)	p-value
MS	9.119 (3.461-24.024)	**<0.001**	9.655 (3.000-31.068)	<0.001
Age (years)	1.023 (0.997-1.050)	0.083		
*BMI ≥ 30 (kg/m²)	8.1 (3.0-21.454)	**<0.001**		
Hypertension	2.656 (1.068-6.604)	0.036		
Dyslipidemia	3.447 (1.417-8.384)	0.006		
Diabetes	2.785 (1.015-7.638)	0.047		
Creatinine (mg/dl)	0.652 (0.162-2.616)	0.546		
*Nitrotyrosine × 100 (μmol/L)	1.136 (1.074-1.202)	**<0.001**	0.64 (0.12-3.52)	0.611
CRP (mg/dl)	1.253 (1.035-1.516)	0.021	0.940 (0.767- 1.151)	0.977
Heart rate baseline (bpm)	1.011 (0.973-1.051)	0.573		
PVC burden (per 100 beats/24 hours increase)	1.017 (0.997-1.038)	0.096		

BMI body mass index; CRP C reactive protein; HR Hazard Ratio; PVC premature ventricular contractions.

*MS, BMI and Nitrotyrosine levels were significantly associated with the study endpoint, P<0.001.

### Complications

After CA 3 hematoma complications, 1 arteriovenous fistula and 1 case of acute pericardial effusion occurred. During the follow up in the catheter ablation failure group, patients remained under AADs and 14 (58,3%) of them experienced a drug related adverse effect: symptomatic low blood pressure in flecainide treated patients (n = 5), obstructive acute lung disease, hypotension and symptomatic bradycardia in beta blockers treated patients (n = 7), and recurrent headaches and hypotension in propafenone group (n = 2).

In this study we have examined the impact of MS on acute and long term follow up catheter ablation in a population of PVCs patients without evidence of structural heart disease and normal heart function. Our main observations were the following: 1. in the overall population who had undergone CA for PVCs, MS was associated with significantly higher acute CA failure. 2. MS was an independent prognostic factor of acute and delayed PVCs recurrence after CA.

MS has some direct or indirect influence on ventricular electrophysiology by different action pathways and altering the ionic channels conduction properties. The higher prevalence of obesity, dyslipidaemia, hypertension and diabetes leads MS patients to an augmented oxidative stress and arrhythmogenesis, with abnormalities in sympatho-vagal balance, QT interval and QT dispersion and a fibrotic and adipotic Cardiac Conduction System involvement [7–10].

The obesity, a common risk factor associated with MS [2], can lead to high rates of sudden cardiac death (SCD), before the development of heart disease [20, 21], and an increased frequency of premature ventricular contractions compared to healthy controls, unrelated to hypertension or concentric ventricular hypertrophy [22, 23].

Elevated plasma free fatty acid level has a stimulatory effect on the sympathetic nervous system [24], with an increased sympathetic and a decreased parasympathetic tone in obese patients [25].

Adipocytokines from epicardial fat significantly decrease delayed rectifier outward currents prolonging action potential duration and facilitating triggered activity with early after depolarizations [9], increasing dispersions of action potential duration and leading to a higher possibility of re-entry circuits [9–11], causing ventricular tachycardia and sudden cardiac death [22]. In our study there is an higher baseline heart rate and PVCs burden in MS than in overall population which may be explained by an abnormal sympatho-vagal balance in MS population, and by a Conduction System involvement with conduction channels alterations and an augmented susceptibility to arrhythmogenesis.

The augmented systemic oxidative stress is associated with cardiac electrical and structural remodelling [26–28], and reactive oxygen species may impair Na, K, Ca channels and Na-Ca exchanger activity, leading to gap junction remodelling, decreasing the action potential amplitude and duration, and increasing the incidence of cardiac arrhythmias in animal models [15]. In fact, oxidative stress results in decreased hERG protein levels, accelerated activation and deactivation of hERG, increased in current amplitude of hERG and hKv1.5, allowing a greater amount of K ions to flow through these channels in the phase 3 of the action potential, down regulation of Ito (transient outward potassium current) responsible for the rapid repolarization phase, and increased the channel opening probability of Ik1 (inward rectifying channel) [27, 28].

The alteration in cardiac oxidative stress, investigated by an higher expression of serum biomarkers like nitrotyrosine and C reactive protein (CRP), leads to alterations in ionic channels conductions properties favouring arrhythmogenic activity in MS population.

At long term these irritative and pro inflammatory stimuli may become pro fibrotic leading to structural fibrotic alterations of the sinoatrial node and throughout the conduction system, the atrioventricular node, atrioventricular bundle and left bundle branch [29]. Added to these fibrotic alterations other structural abnormalities such as localized wall bulging, wall thinning, fatty infiltration, and fibrosis exist in the RVOT, not only in patients with Arrhythmogenic Right Ventricle Cardiomyopathy (ARVC), [30, 31] but also in patients with RVOT tachycardia [32].

If these subclinical myocardial structural alterations may be an important factor limiting the efficacy of CA outcome in general population [33], in MS patients with different morphology of PVCs on surface ECG (different precordial transition and q wave in lead I) and PVCs origin focus have shown not different outcomes, observing all PVCs population and separately RVOT, LVOT and CUSPs PVCs.

This may be explained because, in MS patients, the presence of a constellation of risk factors can alter and affect the prognosis also in evidence of a good ablation target, and in our study the only independent predictor of short and long term follow up catheter ablation failure is MS.

In the two groups there is not a difference regarding the fluoroscopic time and the ablation time (RF time), while there is a significant difference related to total procedural time. We can explain these observations than to the modern technologies utilized, because first in all patients we have utilized as described before two different 3D non fluoroscopic mapping system (CartoXP and Carto 3, Biosense Inc., Diamond Bar, CA; Ensite Navx, St. Jude Medical, Inc., St. Paul, MN, USA), that help to record and target more accurately tachycardia origin for ablation than with fluoroscopy alone, and allow catheter positioning without the use of fluoroscopy, reducing fluoroscopy time and radiation dose [34–36], and secondary also for catheter ablation we can consider to have utilized a standardized protocol, using in more time same energy source (radiofrequency) with same target energy (watt), temperature and pulse duration (maximum 60” for every ablation point). On other hand the higher total procedural time observed in MS may be due to technical difficulties related to multiple risk factors present in MS, than can influence the intraoperative management of MS patients prolonging the total procedural time.

The multifactorial risk factors in MS altering oxido/redux balance by a proinflammatory status, lead to an abnormal sympathetic tone and complex alterations in electrophysiological properties from baseline in affected patients.

All these molecular processes connected with MS represent a pro-inflammatory permanent condition triggering the arrhythmogenic cells firing in patients with PVCs not related to cardiac structural disease and without cardiac dysfunction, and leading to an higher tendency and propensity to arrhythmogenicity.

In a recent study [37] authors have shown a worse catheter ablation outcome in MS patients affected by atrial fibrillation, highlighting the fact that MS related proinflammatory status can affect ablation outcome.

Until now at our knowledge there are not studies focused on idiopathic PVCs catheter ablation outcome in MS overall general population.

In our study we try to show the impact of MS on patients affected by idiopathic PVCs undergoing catheter ablation therapy, without structural heart diseases and altered cardiac function.

The investigated proinflammatory status and altered sympathetic-vagal tone in MS population may identify at baseline patients with less possibility to respond to CA, also in absence of cardiac structural alterations and cardiac dysfunctions and so apparently not different in this from overall population if selected for CA of benign and idiopathic PVCs.

These subclinical alterations are not simple to detect with modern technologies, but in current clinical practice we could at last identify and treat step by step all these risk factors implicated in MS, targeting in this way a better control of a complex clinical condition like MS, before to select MS patients candidates to idiopathic PVCs catheter ablation.

In our opinion next clinical question may be to evaluate in a future prospective randomized trial if a more effective and aggressive control of all these risk factors associated with MS may lead to a better ablation outcome.

This treatment before a catheter ablative approach in MS may be more beneficial and influence short term and long term follow up outcome of idiopathic or benign PVCs .

C. S. received an European Society of Cardiology and European Heart Rhythm Association training grant. S. K. received a Hellenic Cardiological Society training grant. V. K. received a European Society of Cardiology training grant, an European Association of Cardiovascular Imaging research grant.

### Study limitations

Several limitations may have influenced our results. First, although periodic 12-lead ECG and 24 hour Holter were performed during the follow-up period, episodes of asymptomatic PVCs or more serious arrhythmias might have been missed in some patients. Second, the study sample size and follow-up duration may have been insufficient to fully characterize the incidence of lethal arrhythmias among study groups. Third, patients were not followed for any status change in their MS component, that may affect and influence long term ablation outcome. Fourth, we did not obtain waist circumference measurement data, which would have illustrated the abdominal obesity status of the patients, which is considered by some as better tool to assess obesity.

Last, number of subjects and ages in the study and control are not similar.

## Conclusions

The current study demonstrated that after PVCs catheter ablation in structurally and functionally normal ventricles, the patients with MS had significantly higher acute and delayed recurrence rate of PVCs at follow up. Moreover, MS was an independent predictor of arrhythmia recurrence during the follow-up. MS and its proinflammatory status may represent a chronic irritative mechanism in these patients, leading to subclinical alterations that can affect catheter ablation outcomes, with an higher recurrence rate in PVCs mediated via inflammation.

## References

[1] **Third report of the national cholesterol education program (NCEP) expert panel on detection, evaluation, and treatment of high blood cholesterol in adults (Adult Treatment Panel III). Final report** *Circulation* 2002, **106:**3143–3421. 12485966

[2] Ford ES (2004). The metabolic syndrome and mortality from cardiovascular disease and all-causes: findings from the national health and nutrition examination survey II mortality study. Atherosclerosis.

[3] Malik S, Wong ND, Franklin SS, Kamath TV, L'Italien GJ, Pio JR, Williams GR (2004). Impact of the metabolic syndrome on mortality from coronary heart disease, cardiovascular disease, and all causes in United States adults. Circulation.

[4] Alberti KG, Eckel RH, Grundy SM, Zimmet PZ, Cleeman JI, Donato KA, Fruchart JC, James WP, Loria CM, Smith SC, International Diabetes Federation Task Force on Epidemiology and Prevention (2009). Harmonizing the metabolic syndrome: a joint interim statement of the international diabetes federation task force on epidemiology and prevention; national heart, lung, and blood institute; american heart association; world heart federation; international atherosclerosis society; and international association for the study of obesity. Circulation.

[5] Ford ES (2005). Risks for all-cause mortality, cardiovascular disease, and diabetes associated with the metabolic syndrome: a summary of the evidence. Diabetes Care.

[6] Beltrán-Sánchez H, Harhay MO, Harhay MM, McElligott S (2013). Prevalence and trends of metabolic syndrome in the adult U.S. population, 1999-2010. J Am Coll Cardiol.

[7] Soydinc S, Davutoglu V, Akcay M (2006). Uncomplicated metabolic syndrome is associated with prolonged electrocardiographic QTc interval and QTc dispersion. Ann Noninvasive Electrocardiol.

[8] Lee KT, Tang PW, Tsai WC, Liu IH, Yen HW (2013). Differential effects of central and peripheral fat tissues on the delayed rectifier K(+) outward currents in cardiac myocytes. Cardiology.

[9] Corbi GM, Carbone S, Ziccardi P, Giugliano G, Marfella R, Nappo F, Paolisso G, Esposito K, Giugliano D (2002). FFAs and QT intervals in obese women with visceral adiposity: effects of sustained weight loss over 1 year. J Clin Endocrinol Metab.

[10] Marfella R, De Angelis L, Nappo F, Manzella D, Siniscalchi M, Paolisso G, Giugliano D (2001). Elevated plasma fatty acid concentrations prolong cardiac repolarization in healthy subjects. Am J Clin Nutr.

[11] Provotorov VM, Glukhovskiĭ ML (2009). Rhythm and conductivity disorders in patients at the initial stages of metabolic syndrome. Klin Med (Mosk).

[12] Provotorov VM, Glukhovskiĭ ML (2010). Ventricular extrasystole in patients with metabolic syndrome. Klin Med (Mosk).

[13] Gaita F, Giustetto C, Di Donna P, Richiardi E, Libero L, Brusin MC, Molinari G, Trevi G (2001). Long-term follow-up of right ventricular monomorphic extrasystoles. J Am Coll Cardiol.

[14] Lerman BB, Stein KM, Engelstein ED, Battleman DS, Lippman N, Bei D, Catanzaro D (1995). Mechanism of repetitive monomorphic ventricular tachycardia. Circulation.

[15] Dong M, Ren J (2014). What fans the fire: insights into mechanisms of leptin in metabolic syndrome- associated heart diseases. Curr Pharm Des.

[16] Zipes DP, Camm AJ, Borggrefe M, Buxton AE, Chaitman B, Fromer M, Gregoratos G, Klein G, Moss AJ, Myerburg RJ, Priori SG, Quinones MA, Roden DM, Silka MJ, Tracy C, Smith SC, Jacobs AK, Adams CD, Antman EM, Anderson JL, Hunt SA, Halperin JL, Nishimura R, Ornato JP, Page RL, Riegel B, Priori SG, Blanc JJ, Budaj A, European Heart Rhythm Association, Heart Rhythm Society (2006). ACC/AHA/ESC 2006 guidelines for management of patients with ventricular arrhythmias and the prevention of sudden cardiac death: a report of the American college of cardiology/American heart association task force and the European society of cardiology committee for practice Guidelines (Writing committee to develop guidelines for management of patients with ventricular arrhythmias and the prevention of sudden cardiac death). J Am Coll Cardiol.

[17] Wijnmaalen AP, Delgado V, Schalij MJ, Van Taxis CF VH, Holman ER, Bax JJ, Zeppenfeld K (2010). Beneficial effects of catheter ablation on left ventricular and right ventricular function in patients with frequent premature ventricular contractions and preserved ejection fraction. Heart.

[18] Ischiropoulos H (1998). Biological tyrosine nitration: a pathophysiological function of nitric oxide and reactive oxygen species. Arch Biochem Biophys.

[19] Alberti KG1, Zimmet P, Shaw J (2006). Metabolic syndrome--a new world-wide definition. A consensus statement from the international diabetes federation. Diabet Med.

[20] Duflou J, Virmani R, Rabin I, Burke A, Farb A, Smialek J (1995). Sudden death as a result of heart disease in morbid obesity. Am Heart J.

[21] Lalani AP, Kanna B, John J, Ferrick KJ, Huber MS, Shapiro LE (2000). Abnormal signal-averaged electrocardiogram (SAECG) in obesity. Obes Res.

[22] Huang H, Amin V, Gurin M, Wan E, Thorp E (2013). Diet-induced obesity causes long QT and reduces transcription of voltage-gated potassium channels. J Mol Cell Cardiol.

[23] Schunkert H (2002). Obesity and target organ damage: the heart. Int J Obes Relat Metab Disord.

[24] Grekin RJ, Vollmer AP, Sider RS (1995). Pressor effects of portal venous oleateinfusion. A proposed mechanism for obesity hypertension. Hypertension.

[25] Diaz-Melean CM, Somers VK, Rodriguez-Escudero JP, Singh P, Sochor O, Llano EM, Lopez-Jimenez F (2013). Mechanisms of adverse cardiometabolic consequences of obesity. Curr Atheroscler Rep.

[26] Endoh Y, Endoh I, Geczy C, Nakagomi A, Kusama Y (2011). Inflammation and atrial fibrillation. J Arrhythmia.

[27] Drolet B, Simard C, Gailis L, Daleau P (2007). Ischemic, genetic and pharmacological origins of cardiac arrhythmias: the contribution of the Quebec heart institute. Can J Cardiol.

[28] Jeong EM, Liu M, Sturdy M, Gao G, Varghese ST, Sovari AA, Dudley SC (2012). Metabolic stress, reactive oxygen species, and arrhythmia. J Mol Cell Cardiol.

[29] Bharati S, Lev M (1995). Cardiac conduction system involvement in sudden death of obese young people. Am Heart J.

[30] Tandri H, Saranathan M, Rodriguez ER, Martinez C, Bomma C, Nasir K, Rosen B, Lima JA, Calkins H, Bluemke DA (2005). Noninvasive detection of myocardial fibrosis in arrhythmogenic right ventricular cardiomyopathy using delayed-enhancement magnetic resonance imaging. J Am Coll Cardiol.

[31] Nazarian S, Bluemke DA, Halperin HR (2009). Applications of cardiac magnetic resonance in electrophysiology. Circ Arrhythm Electrophysiol.

[32] Globits S, Kreiner G, Frank H, Heinz G, Klaar U, Frey B, Gössinger H (1997). Significance of morphological abnormalities detected by MRI in patients undergoing successful ablation of right ventricular outflow tract tachycardia. Circulation.

[33] Ling Z, Liu Z, Su L, Zipunnikov V, Wu J, Du H, Woo K, Chen S, Zhong B, Lan X, Fan J, Xu Y, Chen W, Yin Y, Nazarian S, Zrenner B (2014). Radiofrequency ablation vs. antiarrhythmic medication for treatment of ventricular premature beats from the right ventricular outflow tract: a prospective randomized study. Circ Arrhythm Electrophysiol.

[34] Sporton S, Earley M, Nathan A, Schilling R (2004). Electroanatomic versus fluoroscopic mapping for catheter ablation procedures: a prospective randomized study. J Cardiovasc Electrophysiol.

[35] Shpun S, Gepstein L, Hayam G, Ben-Haim S (1997). Guidance of radiofrequency endocardial ablation with real-time three-dimensional magnetic navigation system. Circulation.

[36] Smeets J, Ben-Haim S, Rodriguez L, Timmermans C, Wellens H (1998). New method for nonfluoroscopic endocardial mapping in humans. Accuracy assessment and first clinical results. Circulation.

[37] Mohanty S, Mohanty P, Di Biase L, Bai R, Pump A, Santangeli P, Burkhardt D, Gallinghouse JG, Horton R, Sanchez JE, Bailey S, Zagrodzky J, Natale A (2012). Impact of metabolic syndrome on procedural outcomes in patients with atrial fibrillation undergoing catheter ablation. J Am Coll Cardiol.

[CR38] The pre-publication history for this paper can be accessed here: http://www.biomedcentral.com/1471-2261/14/176/prepub

